# Ferroptosis Is a Potential Novel Diagnostic and Therapeutic Target for Patients With Cardiomyopathy

**DOI:** 10.3389/fcell.2021.649045

**Published:** 2021-04-01

**Authors:** Zhenyu Zhai, Pengtao Zou, Fuxiang Liu, Zirong Xia, Juxiang Li

**Affiliations:** Department of Cardiovascular Medicine, The Second Affiliated Hospital of Nanchang University, Nanchang, China

**Keywords:** ferroptosis, regulated necrosis, cardiac damage, cardiomyopathy, heart failure

## Abstract

Cardiomyocyte death is a fundamental progress in cardiomyopathy. However, the mechanism of triggering the death of myocardial cells remains unclear. Ferroptosis, which is the nonapoptotic, iron-dependent, and peroxidation-driven programmed cell death pathway, that is abundant and readily accessible, was not discovered until recently with a pharmacological approach. New researches have demonstrated the close relationship between ferroptosis and the development of many cardiovascular diseases, and several ferroptosis inhibitors, iron chelators, and small antioxidant molecules can relieve myocardial injury by blocking the ferroptosis pathways. Notably, ferroptosis is gradually being considered as an important cell death mechanism in the animal models with multiple cardiomyopathies. In this review, we will discuss the mechanism of ferroptosis and the important role of ferroptosis in cardiomyopathy with a special emphasis on the value of ferroptosis as a potential novel diagnostic and therapeutic target for patients suffering from cardiomyopathy in the future.

## Introduction

The death of myocardial cells is a crucial aspect of cardiac pathophysiology. Damaged cardiomyocytes are eliminated through the activation of six major forms of regulated cell death including necroptosis, ferroptosis, pyroptosis, mitochondrial-mediated necrosis, apoptosis, and autophagic cell death under different conditions (Galluzzi et al., 2018). These regulated myocardial cell death mechanisms participate in the onset and progression of cardiovascular diseases. For example, the mechanism of cardiomyocyte apoptosis has been investigated to a great depth, has been linked to inflammation, infection, ischemia, and immunologically induced damnification in the heart and subsequently heart failure (Kerr et al., 1972; Kang and Izumo, 2003; Wencker et al., 2003; Abbate et al., 2006). Among different necrotic cell deaths, necroptosis contributes significantly to ischemic injuries of the heart, worsening heart function, as well as adverse cardiac remodeling reported by several studies (Luedde et al., 2014; Adameova et al., 2016, 2017; Zhu and Sun, 2018; Ghardashi Afousi et al., 2019). Compared with other forms of myocardial cell death mechanisms, autophagic cell death is not a process that customarily commands the destruction of the cell, it is believed to act as a protective mechanism that recycles the molecular components and unwanted or damaged cellular constituents, thereby maintaining cell vitality. Akazawa et al. (2004) reported that autophagic cell death played a certain part in the pathophysiology of heart failure in transgenic mice. Ferroptosis is another newly identified programmed cell death mechanism that is distinguished from necroptosis and apoptosis; it is iron-dependent and characterized by the toxic lipid reactive oxygen species (ROS) accumulation (Lu et al., 2017), which were also associated with the pathogenesis of several diseases, such as tumors, stroke, ischemia-reperfusion injury, etc. (Guiney et al., 2017; Stockwell et al., 2017). Recently, several studies have demonstrated that ferroptosis played a crucial role in myocardial homeostasis and pathology (Akazawa, 2015; Conrad and Proneth, 2019; Chen et al., 2020; Li W. et al., 2020). However, the biological roles and regulation pathways of ferroptosis in cardiovascular diseases have not been entirely understood.

Ferroptosis is a nonapoptotic, abundant and accessible cellular iron-dependent, and peroxidation-driven programmed cell death pathway, was not discovered until recently with the aid of a pharmacological approach (Dixon et al., 2012). Surprisingly, the erastin and RSL3 induced mode of cell death which was revealed through high-throughput screening of small-molecule libraries, was deemed to be nonapoptotic – as cell death in those treated with erastin and RSL3 occurred without biochemical apoptotic hallmarks. The principle apoptotic machinery with regards to cells treated with erastin and RSL3 – caspases, Bcl-2-associated X protein (Bax) and Bcl-2 homo-logous antago-nist/killer (Bak) – was suppressed in the meantime (Dolma et al., 2003; Yagoda et al., 2007; Yang and Stockwell, 2008; Wolpaw et al., 2011). Further studies identified that the requirement for cellular iron, disruption of the intracellular redox homeostasis controlled by glutathione (GSH), glutathione peroxidase 4 (GPX4), and lipid peroxidation were incorporated in this cell death process (Rui et al., 2020). Recent literature has established key enzymes and metabolites of the ferroptosis pathway and specified chemical modulators (Stockwell et al., 2017). The research about ferroptosis has attached much attention in the context of tumors, pathophysiologically degenerative conditions, and other areas (Guiney et al., 2017; Stockwell et al., 2017; Lin et al., 2020). However, ferroptosis is discovered in cardiac tissue more recently, and there are many studies reported concerning ferroptosis specifically in cardiovascular diseases by using several methods of inducing and inhibiting ferroptosis in cardiac tissue (Baba et al., 2018; Bai et al., 2018; Liu et al., 2018, 2020; Li W. et al., 2019, 2020; Li et al., 2021; Wu et al., 2021). This article will explain the mechanism of ferroptosis and summarized advances of ferroptosis in cardiomyopathy. We hope to deliver novel insights for the research of cardiomyopathy in the future.

## The Mechanism of Ferroptosis

**FIGURE 1:**
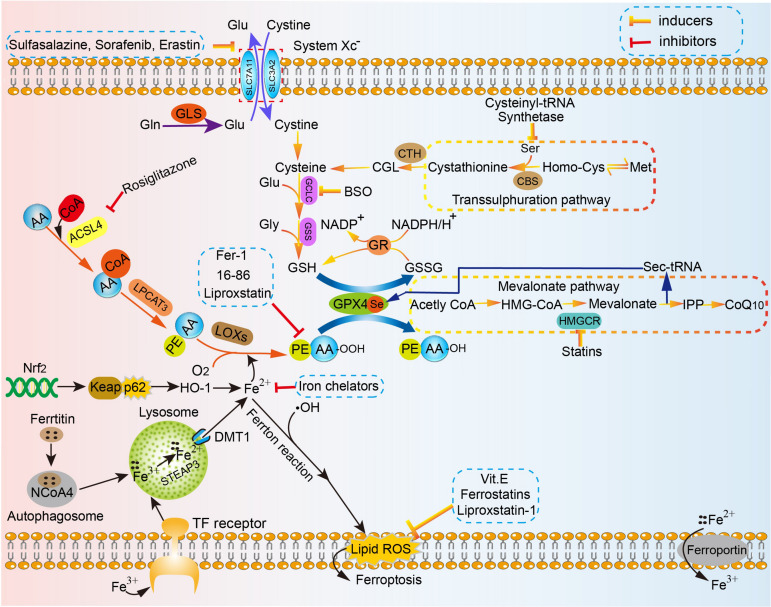
The mechanism of ferroptosis in cell. Amino acid metabolism, which can be affected by GSH consumption and reduced activity and availability of GPX4, iron metabolism, lipid peroxidation metabolism, the high concentration of glutamic acid outside the cell, etc. are strongly implicated in the mechanism of ferroptosis. Homocysteine is converted to cystathionine under the catalysis of the cystathionine b-synthase (CBS), and in the final step, cystathionine is converted to cysteine under the action of the corresponding cystathionine g-lyase in the reverse *trans*-sulfurylation pathway, which can help maintain homeostasis of intracellular cysteine level and subsequently reduce the sensitivity to ferroptotic cell death. The sec-tRNA and CoQ10 produced in the mevalonate pathway can also affect ferroptosis. SLC7A11, the glutamate/cystine antiporter solute carrier family 7 member 11; SLC3A2, the glutamate/cystine antiporter solute carrier family 3 member 2; GSL, glutaminase; Glu, glutamate; Gln, glutamine; GCLC, glutamate-cysteine ligase; GSS, glutathione synthetase; Gly, glycine; GSH, glutathione; GPX4, glutathione peroxidases 4; GR, glutathione reductase; CBS, cystathionine b-synthase; CTH, ceramide trihexoside; Met, Methionine; ACSL4, acyl-CoA synthetase long-chain family member 4; LPCAT3, lysophosphatidylcholine acyltransferase 3; LOXs, lipoxygenases; DMT1, divalent metal transporter 1; ROS, reactive oxygen species; IPP, isopentenyl pyrophosphate; HMGCR, HMG-CoA reductase.

The present definition of ferroptosis is a programmed cell death (PCD) that is reliant on a large number of cellular iron and lipid hydroperoxide, subsequently inducing copious lipid accumulation in cells, interfering with the homeostasis of redox reactions, and eventually promoting cell death (Xie et al., 2016; Dixon, 2017; Imai et al., 2017; Stockwell et al., 2017). This concept distinguishes from canonical signaling cascades for apoptosis or necroptosis, in which the main antioxidant system essentially comprises of various metabolic processes. The associated mechanism of ferroptosis is involved in amino acid metabolism, which was affected by GSH consumption and reduced activity and availability of glutathione peroxidases 4 (GPX4), iron metabolism, lipid peroxidation metabolism, etc. (Figure 1).

### Glutathione Consumption

Xc-system, known as the glutamate-cystine reverse transport system, transports glutamate (Glu) into the extracellular space and, meanwhile, cystine is transported into the cell on an equal ratio. P53 can specifically inhibit Xc-system through down-regulating the expression of SLC7A11 (Kang et al., 2019). The study conducted by Jiang et al. (2015) demonstrated that the antioxidant capacity of human lung cancer H1299 cells remarkably decreased after activation of the P53 gene and cells were prone to ferroptosis (Han et al., 2020). Apart from P53, clinical drugs for cancer cells, including sulfasalazine and sorafenib, and erastin can also induce ferroptosis by inhibiting the activity of cystine/glutamate antiporter Xc-system (Dixon et al., 2012, 2014; Xie et al., 2016). After cystine is transported into the cell, it is converted to cysteine and readily used for GSH production with glutamate-cysteine ligase (GCLC) and glutathione synthetase (GSS) acting as catalysts (Griffith, 1999; Dickinson and Forman, 2002). GSH, as one of major intracellular antioxidant buffers, is widely distributed in tissues of higher organisms. The concentration of intracellular GSH decreases with aging due to variations of numerous factors, including changes in activity of GSH synthetic and metabolic enzymes as well as availability of precursor amino (Jones, 2006). Under normal conditions, intracellular free GSH exists almost exclusively in its reduced form. Reduced GSH is critical in sustaining redox balance under the action of GPX4 *in vivo* (Kalinina and Gavriliuk, 2020). GSH is able to protect important cellular components against damage induced by ROS including free radicals, peroxides, lipid peroxides, and heavy metals (Pompella et al., 2003; Pompella and Corti, 2015). Oxidized glutathione (GSSG) can be converted to free GSH from under the action of GSH reductase. In mammalian cells, the ratio of GSH/GSSG is conservatively estimated at approximately 10,000:1∼50,000:1 within the cytosol under physical condition (Morgan et al., 2013; Lv et al., 2019). The lower ratio of GSH/GSSG, decreasing to values of 10:1 and even 1:1, was observed stimulated by various oxidative stress models (Zitka et al., 2012). The ratio of GSH/GSSG is regarded as one of indicators of oxidative stress in the body (Owen and Butterfield, 2010; Zitka et al., 2012; Sentellas et al., 2014; Kalinina and Gavriliuk, 2020). The finding above was consistent with the results of study by Wu et al. (2018). They found that ferroptosis and mitochondrial dysfunction were induced after co-treatment with 100 μM t-BHP for 1 h in PC12 cells, which was a widely used oxidative stress stimulus, accompanied by GSH depletion, decrease of the ratio of GSH/GSSG, reduced Gpx4 expression, and increased lipid ROS (Wu et al., 2018). The results of study by Wu et al. (2018) indicated that the ratio of GSH/GSSG is tightly related to ferroptosis as well as GSH depletion. Importantly, Sun et al. (2018b) conducted the study to explore the association between GSH consumption and ferroptosis for the first time, and the results demonstrated that GSH deletion could trigger ferroptosis by generating lipid peroxidation build-up in retinal pigment epithelial (RPE) cells (Qiu et al., 2020). Therefore, it can be concluded that GSH consumption is regarded as an indispensable process leading up ferroptosis.

The upstream factors mediating deprivation of intracellular GSH can be summarized in three aspects: glutamine (Gln) decomposition, reduced concentration of cysteine and high concentration of extracellular glutamate. More than half of the free amino acids in the human body are in the form of glutamine in muscles and other tissues. Extracellular glutamine could be transformed to Glu, under the action of glutaminase 1 (GLS1) and glutaminase 2 (GLS2), which is then converted to a-ketoglutarate (a-KG) by using the deamination reaction. In a final step, a-KG is degraded by the mitochondrial tricarboxylic acid cycle (TCA). The research proposed by Gao M. et al. (2019) demonstrated that knockdown of GLS2 to inhibit glutamine decomposition pathway could suppress ferroptosis. Cells can maintain levels of intracellular cysteine by the glutamate–cystine reverse transport system as described earlier, which can offer the oxidized form of cellular cysteine–cystine, and the reverse *trans*-sulfurylation pathway, which can convert methionine to homocysteine, cystathionine, in turn, and eventually to cysteine (Hayano et al., 2016). Factors contributing to decreased availability of cysteine greatly promoted the occurrence of ferroptosis. The glutamate/cystine antiporter solute carrier family 7 member11 (SLC7A11), cationic amino acid transporter, promotes the synthesis of GSH by mediating cystine uptake and Glu release, protects cells from oxidative stress, maintains cell redox balance, and prevents lipids peroxidation induced ferroptosis. The study by Jiang et al. (2015) indicated that p53 could limit the availability of cysteine via suppression of SLC7A11 expression, making cells prone to ferroptosis. Cysteinyl-tRNA synthetase encoded by the CARS gene is associated with protein translation of tRNAs by using cysteine. The conclusion has been proven by Hayano et al. (2016) that the knockout of *CARS* gene by three additional Ambion Silencer Select siRNA sequences could make the increase of intracellular free cysteine, preventing Erastin-induced ferroptosis. Homocysteine is converted to cystathionine under the catalysis of the cystathionine b-synthase (CBS), and in the final step, cystathionine is converted to cysteine under the action of the corresponding cystathionine g-lyase in the reverse *trans*-sulfurylation pathway. In a study to explore the influence of the reverse *trans*-sulfurylation pathway on the resistance of drugs, the researchers discovered that sensitivity of these resistant cells to ferroptosis might be restored through upregulating the pathway, whereas the pathway cannot prevent Ras-selective lethal small molecule 3 (RLS3), the GPX4 inhibitor, induced ferroptosis due to acting on the “upstream” of ferroptosis (Hayano et al., 2016). In addition, when the concentration of extracellular glutamate increases abnormally, the concentration gradient of glutamate inside and outside the cell changes, subsequently affecting the cellular exchange of Glu and cystine in a 1:1 manner, ultimately leading to lipid peroxidation accumulation, and consequently ferroptosis (Yang and Stockwell, 2016; Yang et al., 2016; Latunde-Dada, 2017). Studies have indicated that oxidation toxicity mediated by high extracellular glutamate could induce nerve cell injury manifesting as ferroptosis (Yang et al., 2014; Zheng et al., 2017), and the results of the research conducted by Liu further support the view above (Liu et al., 2017). In a nutshell, changes based on Gln decomposition, reduced availability of cysteine and high concentration of extracellular glutamate can silence the Xc-system, causing GSH- consumption induced ferroptosis.

### Decreased Activity or Availability of GPX4

If GSH metabolism is the crux of the amino acid metabolism mechanism of ferroptosis, GPX4 is the channel that join all the modifications. GPX4, as a crucial antioxidant enzyme, is different from the other GPX family members in the fields of its monomeric structure, a less restricted dependence on GSH as reducing substrate, and the ability to reduce lipid-hydroperoxides inside biological membranes. Reduced GSH is converted to the oxidized form of glutathione (glutathione disulfide, GSSG), which is recycled by GSH reductase and NADPH/H^+^, under the catalysis of GPX4 during the reduction of hydrogen peroxide, organic hydroperoxides, and lipid peroxides, protecting cells from oxidative stress. GPX4, the GSH-dependent antioxidant enzyme, can reduce lipid hydroperoxides (PUFAs-OOH) to the corresponding alcohol by using two units of GSH as a donor, inhibiting the oxidative stress induced ferroptosis (Lv et al., 2019). Inactivation of the system XC (−)/glutathione/glutathione peroxidase 4 (Gpx4) axis can bring about an accumulation of lipid peroxides, subsequently leading to ferroptotic cell death (Friedmann Angeli et al., 2014; Yang et al., 2014). The factors contributing to reduced activity or availability of GPX4 will increase oxidative stress and make cells prone to the occurrence of ferroptosis. At present, specific ferroptosis-inducing agents included Erastin, RSL3, and ferroptosis-inducing agents 56 (FIN56) (Yang and Stockwell, 2008; Shimada et al., 2016). In particular, RSL3 and FIN56 are usually used to trigger ferroptosis by limiting the activity or availability of GPX4. Yang et al. (2014) found that RSL3 through the mechanism silencing GPX4 could increase oxidative stress, resulting in ferroptosis. Moreover, the research led by Shimada et al. (2016) has demonstrated that FIN56 can reduce GPX4 abundance by consuming GPX4 protein. Moreover, the study conducted by Dabkowski et al. (2008) reported that, for the first time, mitochondria-specific transgenic overexpression of GPX4 could attenuate myocardial ischemia/reperfusion (I/R)-associated cardiac contractile dysfunction, which was relevant to enhanced mitochondrial electron transport chain (ETC) complex activities.

Recently, ferroptosis suppressor protein 1 (FSP1), which was previously known as apoptosis-inducing factor mitochondrial 2 (AIFM2), is regarded as another potent factor to protect cells against ferroptosis.

Bersuker et al. (2019) reported that myristoylation was capable of recruiting FSP1, which was identified through a synthetic lethal CRISPR–Cas9 screen, to the plasma membrane and reduced coenzyme Q10 (CoQ_10_) as an oxidoreductase, subsequently keeping lipid peroxides from propagation within membranes in the absence of GPX4. The results of the study demonstrated that a novel ferroptosis suppression pathway tightly related to FSP1 acted in parallel to the canonical GSH-based GPX4 pathway. The findings by Bersuker et al. (2019) was consistent with, to some extent, the results of the study conducted by Doll et al. (2017, 2019). They also found that FSP1 was able to protect cells from ferroptosis induced by GPX4 depletion in a cDNA overexpression screen complementing for *GPX4* loss and CoQ_10_, known as ubiquinone, could be regenerated under the catalysis of FSP1 using NAD(P)H. CoQ_10_ was capable of scavenging small-molecule lipophilic radical, such as ferrostatin-1 (Fer-1) and liproxstatin-1, leading to halting ferroptosis (Dixon et al., 2012; Bebber et al., 2020). The findings above indicated that pharmacological inhibition of FSP1 may be an effective therapeutic method to sensitize cancer cells to ferroptosis-inducing chemotherapeutic agents.

### Lipid Peroxidation Metabolism

Recent evidence has demonstrated that lipid peroxidation metabolism is associated with the form of ferroptosis, which participates in the establishment of membranous micelles and pores (Borst et al., 2000; Yang et al., 2016; Doll et al., 2017; Agmon et al., 2018). At present, it is believed that the formation of lipid hydroperoxides is related to lipoxygenase (LOXs)-catalyzed autoxidation and enzymatic reactions rather than cyclooxygenases (COXs) (Yang et al., 2016). Currently, studies on lipid peroxidation metabolism related to ferroptosis mainly focus on enzyme-catalyzed lipid peroxidation reactions. Under ferroptosis, the peroxidation of polyunsaturated fatty acids (PUFAs) seems to be mainly regulated by LOXs and GPX4 (Seiler et al., 2008; Yang et al., 2016). In particular, LOXs, which are iron-containing nonheme dioxygenases, directly catalyze lipid peroxidation by promoting the di-oxygenation of free and esterified PUFAs (Kuhn et al., 2015), whereas GPX4 indirectly inhibits lipid peroxidation (Seiler et al., 2008). Friedmann Angeli et al. (2014) found that multiple LOXs were associated with PUFA peroxidation and under GPX4 inactivity the accumulation of oxidized PUFAs could make cells to occur ferroptosis (Dixon et al., 2012). Additionally, ROS scavengers, such as liproxstatin-1 (Lip-1), ferrostatin-1 (Fer-1), as well as coenzyme Q10, vitamin E and their analogs can inhibit the lethal cascade related to ferroptosis (Yagoda et al., 2007; Friedmann Angeli et al., 2014; Matsushita et al., 2015; Kagan et al., 2017; Zilka et al., 2017). The metabolism of arachidonic and adrenic acids, which are groups of PUFAs, are associated with two important enzymes – acyl-CoA synthetase long-chain family member 4 (ACSL4) and lysophosphatidylcholine acyltransferase 3 (LPCAT3), both of which engage with the incorporation of long PUFAs into lipid membranes, and several studies have proven that inhibition of ACSL4 and 3 LPCAT3 by genetic and/or pharmacological can protect cells from ferroptosis in some settings (Dixon et al., 2015; Yuan et al., 2016b; Doll et al., 2017; Kagan et al., 2017). PUFAs can be converted to PUFA-CoA under the catalysis of acyl-CoA synthase. Arachidonic acid (AA) is usually preferentially thioesterified under the action of ACSL4, subsequently are involved in the formation of phospholipids, when oxidized, it forms phosphatidy-lethanolamine to make cells prone to ferroptosis in a final step (Golej et al., 2011). Doll et al. (2017) has highlighted that inactivating ACSL4 gene and pharmacologically inhibiting ACSL4 with distinct thiazolidinediones (TZDs), namely rosiglitazone (ROSI), pioglitazone (PIO) and troglitazone (TRO), can effectively obstruct ferroptosis as this hinders the assembly and movement of PUFAs-OOH, indicating that Acsl4 inhibition is a viable therapeutic method to prevent diseases related to ferroptosis.

Although enzyme-catalyzed lipid peroxidation reactions have become the focus of many researchers, it is also essential not to ignore the importance of non-enzymatic lipid peroxidation. The progression of oxygen-driven free radical chain reaction, namely non-enzymatic lipid peroxidation, includes three main processes in turn (Frank, 1950). Initiation refers to the generation of early lipid radical L⋅, since a hydrogen atom is pumped away from the lipid molecule LH under the premise of generating a large number of sufficiently reactive free radicals such as ⋅OH; During the next step propagation, L⋅ undergoes a series of stages including hydrogen pumping, addition, fracture, etc. This process continuously repeats to produce a chain reaction. The oxidation process will not be stopped as long as the reaction remains dominant. The progress of termination occurs with a limited amount of antioxidants acting as free radical scavengers, and eventually, the reaction slows down and becomes terminated. Moreover, lipid molecules are constantly recruited to free radical reactions by PLOO⋅ and PLO⋅ produced through the spontaneous oxidation of lipid peroxidation (Davies and Guo, 2014). Fenton reaction discovered in 1984 by H. J. H. Fenton is currently believed to be the provider of free radicals for lipid peroxidation metabolism, so are the Fenton-like reaction (Lai and Piette, 1978).

### Iron Metabolism

Iron is known as one of important trace elements for cell survival in the body, the majority of which is distributed in cells and stored in ferritin and incorporated into heme and iron-sulfur (Fe-S) cluster proteins (Wang and Pantopoulos, 2011). Iron is closely related to a variety of biological processes under physiological states, such as delivering oxygen to cells by binding to heme for cellular generation of ATP and that is energy metabolism, deoxyribonucleic acid (DNA) synthesis and repair, cellular respiration, and electron transfer, participation in redox reactions, and the generation (Fe-S) protein clusters which can regulate gene expression, as well as overall metabolism (Johnson et al., 2005; Pantopoulos et al., 2012; Hirst, 2013; Lawen and Lane, 2013; Abbaspour et al., 2014; Loreal et al., 2014; Sumneang et al., 2020). Similar to other cell types, the endogenous levels of iron concentration in cytosol, mitochondria, nuclei, or lysosomes within cardiomyocytes is approximately 6, 16, 7, and 16 μM, respectively, under normal conditions (Petrat et al., 2001; Rauen et al., 2007; Nakamura et al., 2019; Sumneang et al., 2020). There is only one pathway for iron export from cardiomyocytes and that is through Fpn1. Nevertheless, iron is able to enter cardiomyocytes through several ways, which makes cardiomyocytes particularly prone to iron overload under pathological conditions. A detailed discussion with regard to the mechanism of cellular iron regulation in the heart has been reviewed elsewhere (Abbaspour et al., 2014; Gao G. et al., 2019; Ghafourian et al., 2020; Ravingerova et al., 2020). Moreover, cellular excess iron makes cardiomyocytes vulnerable to ferroptosis through the production of ROS. There exists a close relationship between the ferroptosis and the homeostasis of iron metabolism in cells.

Gao et al. (2015) confirmed the significance of iron in the formation of ferroptosis through experimental methods and established that cells became more susceptible to ferroptosis after a rise in iron level within the redox-active labile iron pool (LIP) (Hou et al., 2016). The study by Dixon et al. (2012) indicated that reduce iron in LIP by several methods could suppress the formation of ferroptosis (Kwon et al., 2015). Notably, ferritinophagy is a critical mechanism to regulate the level of LIP. LIP is composed of ferrous iron in a soluble, chelatable state within the cytoplasm and is regulated by ferritin, the substrate of ferritinophagy, which is a highly conserved iron storage protein and is made up of two subunits including H-ferritin and L-ferritin (Cordani et al., 2019; Zhang et al., 2019). Ferritinophagy is a process that ferritin is sequestered into autophagosomes and delivered to lysosomes for degradation and is important for maintaining iron homeostasis in cells (Kidane et al., 2006; Asano et al., 2011; Mancias et al., 2014; Masaldan et al., 2018). Previous studies have reported that iron chelator, such as DFO and DpdtC, is capable of inducing ferritinophagy (Mancias et al., 2014; Huang et al., 2018). The study conducted by Gao et al. (2015) demonstrated that ferritinophagy could trigger ferroptosis by promoting the accumulation of iron and ROS, which was consistent with the finding by Hou et al. (2016) and Tang et al. (2018). Controlling iron level in cells by interrupting ferritinophagy may be a new therapeutic target for inhibiting ferroptosis in the future (Sui et al., 2019; Li N. et al., 2020). However, the human body can sustain iron homeostasis in both the cell and the whole by several proteins and pathways, such as the iron-responsive element (IRE)-binding proteins, also known as IRE-BP, IRBP, IRP and IFR, which attach to IREs during the regulation of iron metabolism within human bodies (Gray and Hentze, 1994; Eisenstein, 2000). In addition, transferrin, which is an important carrier glycoprotein of serum iron that becomes endocytosed into cells through transferrin receptor (TFRC). Both transferrin and its receptor are perceived as important participants of regulating iron metabolism (Gao et al., 2015). Yang and Stockwell (2008) found that increasing unstable iron intake by upregulating TFRC could increase the sensitivity of cells toward ferroptosis. Iron is a central co-factor for several molecules and enzymes and is particularly involved with regulating mitochondrial function (Levi and Rovida, 2009; Stehling and Lill, 2013). In the context of cardiomyocyte, mitochondria is predominantly crucial for sustaining the normal functions of cardiomyocyte, hence further highlighting the indispensable role of iron during cardiac function, since mitochondria fuel the cardiac muscles to constantly contract (Barth et al., 1992). Disturbance of iron homeostasis including iron deficiency and accumulation of iron can impair the normal cardiac function and result in various cardiovascular diseases (von Haehling et al., 2015; de Montalembert et al., 2017; Fujikura et al., 2018; Lakhal-Littleton, 2019). Excess iron can be transported and accumulated into cardiac tissue and cardiomyocytes from iron-overload disorders or other cardiac pathologies (Oudit et al., 2003). Accordingly, excess iron will cause the overproduction of mitochondrial ROS. When the surplus of iron is also taken into the mitochondria, it consequently becomes a hotbed of ROS production from oxidation phosphorylation and H2O2 production (Oudit et al., 2003; Levi and Rovida, 2009; Bolduc et al., 2019; Fang et al., 2019; Gao M. et al., 2019). In addition, Fe^3+^ can be converted to Fe^2+^, under the action of the metal reductase STEAP3, and then divalent metal transporter 1 (DMT1) releases Fe^2+^ in lysosome into cytoplasmic LIP through. These soluble, redox-active free iron in the LIP is considered as the catalyst that induces the elevated ROS production in ferroptosis, thereby causing cardiomyocytes more sensitive to oxidative stress in the presence of excess iron (Thomas et al., 2013; Melenovsky et al., 2017; Xu et al., 2019).

### Other Pathways Related to Ferroptosis

Apart from GSH consumption, reduced activity and availability of GPX4, lipid peroxidation metabolism, iron metabolism, and other pathways are also correlated with the mechanism of ferroptosis; for instance, organelle-mediated pathways, Nrf2 pathway, TP53 pathway, etc. Importantly, Statin drugs is capable of making cells vulnerable to ferroptosis through inhibiting the rate-limiting enzyme of the mevalonate pathway, HMG CoA reductase, presumably by depleting CoQ10 and possibly by also inhibiting downstream tRNA isopentenylation via TRIT1, which is necessary for the biosynthesis of GPX4 (Fradejas et al., 2013; Shimada et al., 2016; Viswanathan et al., 2017). Moreover, several cell structures including mitochondria, endoplasmic reticulum (ER), lysosomes are involved in the formation of ferroptosis by mediating multiple pathways. The research led by Gao M. et al. (2019) have confirmed that mitochondria have a central role in ferroptosis, which can affect the pathway of glutamine decomposition and subsequently result in ferroptosis (Tadokoro et al., 2020). Yuan et al. (2016a) also discovered that inhibition of CDGSH iron sulfur domain 1 (CISD1), which is an iron sulfur protein that can suppress iron transportation during the aforementioned progression, could prevent lipid peroxidation and ferroptosis by suppressing mitochondrial iron uptake through RNAi technology or pioglitazone pharmacology. The evidence indicated that ER oxidative stress markers that activate cation transport regulator homolog 1 (CHAC1), transcription factor 4 (ATF4), and phosphorylation of eIF2a were all upregulated during ferroptosis (Dixon et al., 2014). However, the precise correlation between ER and ferroptosis still remains vague, and further research is needed to explore its function in ferroptosis. Recent researches suggest that lysosomes are also related to ferroptosis. Mancias et al. (2014) found that the cargo receptor NCOA4 transferring ferritin to lysosomes also participates in ferroptosis. In addition, the study by Hou et al. (2016) proposed that the knockout of autophagy-related genes Atg5 and Atg7 also limits Erastin-induced ferroptosis in cells, since ferroptosis is dependent on autophagy. Abdalkader et al. (2018) found multiple genes controlled by the transcription factor nuclear factor erythroid 2-related factor 2 (Nrf2) were involved in ferroptosis, such as GCLM, GSS, SLC7A11, MT1G, TFRC, and so on. The study conducted by Jennis et al. (2016) indicated that the up-regulated GLS2 targeting for TP53 (p53 genes) could result in p53-dependent ferroptosis.

## The Role of Ferroptosis in Cardiomyopathy

Cardiomyopathy is closely related to the progress of heart failure, especially lethal heart failure, such as diabetic cardiomyopathy (DCM), doxorubicin (DOX)-induced cardiotoxicity, dilated cardiomyopathy, hypertrophic cardiomyopathy, and so on (Maron et al., 2018; Fang et al., 2019; Rosenbaum et al., 2020; Wei et al., 2020). Loss of terminally differentiated cardiomyocytes is identified as a principle risk factor in the onset of multiple cardiomyopathies. However, the mechanism of cardiomyocyte death has not been completely unveiled. Literature has indicated that the newly discovered iron-dependent ferroptosis is implicated in many cardiomyopathies including ischemia/reperfusion (I/R)- and DOX-induced cardiomyopathy (DIC), iron overload cardiomyopathy (IOC), DCM, septic cardiomyopathy, etc. The focus of this study will be on the role of ferroptosis in the pathophysiology of the four kinds of cardiomyopathy above, and we hope to provide a fresh insight for the diagnosis and treatment of cardiomyopathy.

### Ferroptosis and Doxorubicin-Induced Cardiomyopathy

There are two main classifications for DOX-induced myocardial injuries: contractile dysfunction and loss of myocyte. Though both are deemed crucial in the progression of DIC, loss of myocyte could be more important as it is an irreversible process and generates a poorer prognosis, even fatal decades after onset (Felker et al., 2000). However, the mechanisms that lead to cardiomyocyte death are not fully understood. Fang et al. (2019) proposed that ferroptosis, which is an iron-dependent, and peroxidation-driven programmed cell death form, was observed in the murine model of DIC, and suppression of ferroptosis by ferrostatin-1 (Fer-1) substantially alleviated DIC. The outcomes of the study also revealed hemeoxygenase-1 (Hmox1), which is widely acknowledged for its robust cardioprotection (Yet et al., 2001; Wang et al., 2010), was significantly stimulated in heart induced by DOX, and free iron released on heme degradation by Nrf2-mediated up- regulation of Hmox1 was necessary for inducing cardiac injury. Importantly, Fer-1 and iron chelation also alleviated both acute and chronic I/R induced heart failure in mice models. The results above were consistent with the finding by Liu et al. (2020) that Fer-1was capable of inhibiting ferroptosis, subsequently preventing cardiac injury, along with the ultrastructural changes of cardiomyocyte mitochondria. The study conducted by Liu et al. (2020) also showed ferroptosis was a crucial mechanism in DIC and highlighted the crucial role of Acyl-CoA thioesterase 1 (Acot1) during the process, which was related to its biochemical function by shaping the lipid composition, indicating that Acot1 bears the potential of becoming a therapeutic target in preventing DIC by inhibition of ferroptosis. The findings by Tadokoro et al. (2020) suggested that mitochondria-dependent ferroptosis played an important role for cardiomyopathy induced by DOX (DIC) in the mice model via downregulated glutathione peroxidase 4 (GPX4) and excessive lipid peroxidation caused by DOX through DOX-Fe^2+^ complex in mitochondria, which could be reversed by GPX4 overexpression or iron chelation targeting Fe^2+^ in mitochondria in cardiomyocytes. They also reported that Fer-1 and zVAD-FMK, which were concomitant inhibitors of ferroptosis and apoptosis, were capable of fully protecting cardiomyocytes against death induced by DOX (Tadokoro et al., 2020). These researches emphasize that targeting ferroptosis would be a reasonable protective approach for preventing DIC. Interestingly, although Fang et al. (2019) demonstrated that ferroptosis induced by iron overload is a major pathogenesis factor of the DIC, knocking out receptor interacting serine/threonine kinase 3 (Ripk3) could increase survival rates compared with Fer-1 treatment alone, which indicated that ferroptosis and necroptosis were simultaneously involved in tissue damage as researchers had reported before (Linkermann et al., 2014). The link between ferroptosis and necroptosis warrants further investigation in DIC.

### Ferroptosis and Iron Overload Cardiomyopathy

At the cellular level, iron is involved in multiple biochemical reactions, which serves as crucial component of a variety of enzymes participating energy metabolism, cellular respiration, synthesis and repair of DNA (Lawen and Lane, 2013; Loreal et al., 2014). However, excessive iron accumulation, namely iron overload, in cells is an important implication of several diseases disrupting the homeostatic systemic iron regulatory mechanism, such as primary hemochromatosis and transfusion-dependent anemia (Gujja et al., 2010; Gao et al., 2014; Kontoghiorghe and Kontoghiorghes, 2016). Importantly, iron overload in cardiomyocyte can result in IOC, the major reason of fatality in patients suffering from hemochromatosis. IOC manifests as progressive electromechanical deterioration of the heart (Nakamura et al., 2019). It is well known that the lethal level of lipid peroxidation is the significant feature of ferroptosis, which can be influenced by several factors, such as ROS, lipoxygenases (LOX), cyclooxygenases (COX), and GPX4. Hence, excessive accumulation of ROS, enhanced activities of LOX and/or COX, and decreased activity or availability of GPX4 are capable of inducing irresistible lipid peroxidation, subsequently resulting in ferroptotic cell death (Friedmann Angeli et al., 2014; Yang et al., 2014; Galluzzi et al., 2018; Lei et al., 2019). In this regard, iron overload in cardiomyocyte is able to trigger ferroptosis through several means, including using the Fenton reaction to catalyze the reactions for ROS production, and serving as a co-factor for LOX, allowing this enzyme to oxidize PUFAs, indicating that ferroptosis may be tightly related to IOC (Friedmann Angeli et al., 2014; Yang et al., 2014; Baba et al., 2018; Galluzzi et al., 2018; Lei et al., 2019). However, the mechanism underling how iron overload leads to IOC has not been fully elucidated and more studies need to be conducted to investigate the role of ferroptosis in IOC in the future (Ravingerova et al., 2020; Sumneang et al., 2020). In addition, Baba et al. (2018) reported that erastin (8 μM), RSL3 (1 μg/ml), and isoprenaline (1 μM), which were specific ferroptosis-inducing compounds, could lead to ferroptosis by reducing GSH availability, suppressing GPX4 activity, and interfering with many of the molecules involved in regulating iron concentration and iron-mediated redox reactions, such as GPX4, NADPH oxidase 4 (Nox4), and ferritin heavy chain (Liu et al., 2018). Ferroptosis in cardiomyocyte can be inhibited by ferrostatin-1, increased mechanistic rapamycin signaling target (mTOR), overexpression of ectonucleotide pyrophosphatase/phosphodiesterase family member 2 (ENPP2), and administration of puerarin (Baba et al., 2018; Bai et al., 2018; Liu et al., 2018).

### Ferroptosis and Diabetic Cardiomyopathy

Diabetic cardiomyopathy, which manifests as hypertrophy and fibrosis in the heart, can result in early ventricular diastolic dysfunction and late ventricular systolic dysfunction in a chronological order without changes in blood pressure and coronary disease in clinic (Bugger and Abel, 2014; Kurmus et al., 2018; Parim et al., 2019), which is perceived as one of the most common complications of diabetes that is linked with increased risk of heart failure (Paolillo et al., 2019). DCM is a kind of multifactorial disorder, and the mechanism of DCM are involved in insulin resistance, hyperglycemia, oxidative stress, fatty acids, myocardial fibrosis, inflammatory response, mitochondrial dysfunction, hypertrophy, ER stress, etc. (Wang et al., 2014; Cao et al., 2015; Chen et al., 2018). Basically, the terminal pathway of cardiomyocytes during DCM is cell death. Many studies have found that myocardial cell death patterns in DCM include four types, such as apoptosis, autophagy, necrosis, and entosis (Martins et al., 2017; Tang et al., 2019). More recent researches have elucidated that ferroptosis, which is a recently discovered form of cell death first proposed by Dixon et al. (2012), is linked with the pathological progress of DCM. Oxidative stress, which can interfere the balance between antioxidant capacity and the production of ROS, has been widely accepted as a common mechanism of DCM (Khullar et al., 2010; Huynh et al., 2014). Considering that ROS formation promotes ferroptosis, it is very likely that ferroptosis is involved in DCM (Anandhan et al., 2020; Chen et al., 2020). Zang et al. (2020) showed that diabetes was capable of inducing autophagy deficiency with time, resulting in Nuclear factor-erythroid factor 2-related factor 2 (Nrf2)-mediated defense was turned off. Subsequently, Nrf2-operated pathological program was turned on, which made cells prone to ferroptosis, leading to worsening the progression of DCM (Zang et al., 2020). This indicated that more attention should be given with regard to ferroptosis mediated by the Nrf2 pathway. A growing number of evidence showed that Nrf2 and its target genes, which possessed the anti-oxidant, anti-inflammatory, anti-apoptotic, anti-ferroptotic, and anti-fibrotic functions, could protect β islet cells of the pancreas against the oxidative damage induced by high glucose in DCM. Studies demonstrated that many natural and synthetic activators of Nrf2 might have the promising therapeutic values on DCM in in animal models of DCM (Ge et al., 2019). Pharmacological inhibition of Nrf2-mediated pathway may be a therapeutic target for preventing DCM in the future. Bruni et al. (2018) reported that β islet cells of the pancreas in the body were vulnerable to ferroptosis induced by erastin or RSL3, and the damage to the human β islet cells could be reversed by Fer-1. However, they also found that the function of β islet cells, which were treated with erastin, RSL3, or both compounds, were not weakened before transplantation into an immunodeficient recipient mouse, indicating that the relation between ferroptosis and the dysfunction of β islet cells needs further investigation. Moreover, Li W. et al. (2020) indicated that ferroptosis was implicated in the ischemia/reperfusion injury of DCM through endoplasmic reticulum stress (ERS), which is a cellular response to ER dysfunction and can be induced by ROS, and suppression of ferroptosis could alleviate diabetes mellitus myocardial ischemia/reperfusion injury (DIR), which may provide a new therapeutic target for DCM.

### Ferroptosis and Septic Cardiomyopathy

Septic cardiomyopathy is a kind of reversible complication in patients suffering from sepsis, and is also one of the major causes of high mortality of sepsis (Zechendorf et al., 2020). A prominent feature in the progress of septic cardiomyopathy is death of terminally differentiated myocardial cells. Previous studies demonstrated that lipopolysaccharide (LPS) or stimulator of interferon genes (STING) were closely implicated in sepsis-induced cardiac dysfunction by causing apoptosis, autophagy, pyroptosis, or cardiomyocytes necroptosis (Suzuki et al., 2003; Wang et al., 2015; Sun et al., 2018a; Li N. et al., 2019). Nevertheless, evidence shows that other kinds of cell death may be part of the pathogenesis of septic cardiomyopathy, because the suppression of autophagy, pyroptosis, or apoptosis alone is able to partially relieve the sepsis-induced cardiac injury (Suzuki et al., 2003; Sun et al., 2018a; Li W. et al., 2019). The higher expression level of cyclooxygenase-2 (COX-2), also known as prostaglandin endoperoxide synthase 2 (PTGS2) – a recognized marker of ferroptosis – was observed in the heart of murine model with sepsis (Shen et al., 2007; Frazier et al., 2012; Yang et al., 2014). In addition, mitochondria changes in myocardial cell damage induced by LPS were consistent with mitochondrial characteristics of ferroptosis in cardiomyocytes (Xie et al., 2016; Sun et al., 2018a). The findings above indicated that ferroptosis may be closely linked to the progression of septic cardiomyopathy induced by LPS. Importantly, the study conducted by Li W. et al. (2020), which aimed to investigate the role and underlying mechanism of ferroptosis on septic cardiac injury induced by LPS, demonstrated that LPS was able to promote the expression of nuclear receptor coactivator 4 (NCOA4) but decrease the level of ferritin, which was degraded in a ferritinophagy-dependent manner through the interaction between NCOA4 and ferritin, leading to a higher level of Fe^2+^ was released into cytoplasm. Subsequently, the expression of siderofexin (SFXN1) on mitochondrial membrane was activated by Cytoplasmic Fe^2+^, which in turn transported cytoplasmic Fe^2+^ into mitochondria, resulting in the accumulation of mitochondrial ROS and making cardiomyocyte sensitive to ferroptosis. This indicated that ferroptosis mediated by ferritinophagy could confer damage upon cardiomyocyte for sepsis-induced cardiac injury. The results of the study were consistent with the discovery of previous research that mitochondrial iron reduction could avert cardiac ischemic damage through suppressing mitochondrial ROS production (Chang et al., 2016; Sumneang et al., 2020). Therefore, aiming ferroptosis in cardiomyocyte could be a novel clinical approach of treating cardiac injury induced by sepsis.

## Summary and Prospect

This review has outlined our knowledge about the mechanism of ferroptosis, and described the role of ferroptosis in cardiomyopathy. An emphasis in the duality of ferroptosis, including amino acid metabolism and iron-overload counterparts, is evident throughout the manuscript, derived mostly from recent studied aiming to investigate the role and underlying principles of ferroptosis on cardiomyopathy.

However, apart from amino acid metabolism and iron metabolism, lipid peroxidation metabolism, the high concentration of glutamic acid outside the cell, organelle-mediated pathways, Nrf2 pathway, TP53 pathway, etc. are also implicated in the mechanism of ferroptosis. Oxidative stress, which is the final downstream event of the pathway related to ferroptosis, could be induced either by way of a lack of enzymatic antioxidants, or loss of iron homeostasis, subsequently causing ferroptosis. Ferroptosis was also involved in pathological cell death of other diseases of cardiovascular system, for instance heart failure and myocardial infarction (Liu et al., 2018; Chen et al., 2019; Park et al., 2019; Yoshimura et al., 2020). However, there is no study conducted to explore the relationship between ferroptosis and the pathogenesis of arrhythmia, such as ventricular tachycardia, atrial fibrillation, and ventricular fibrillation. Interestingly, to identify atrial fibrillation (AF) -related mRNAs, we collected human right atrial appendage tissues from five patients suffering persistent AF (AF group) and five patients with normal sinus rhythm (NSR group) and characterized the global changes in mRNA expression with high-throughput sequencing technology. We found that SLC7A11 was significantly downregulated (the results of our study have not been published), which is a cystine/glutamate transporter gene, a key gene regulating “iron overload-mediated ferroptosis,” and an important part of the amino acid reverse transport system (Fang et al., 2020). Additionally, further research is called for to clarify the mechanism of triggering of ferroptosis at the molecular level in various chronic and acute cardiovascular system disorders; and whether there exists any difference in the regulation of ferroptosis based on myocardial cell type, patient age, and other factors. To respond to the questions above, it is imperative to establish methods to identify the specific cells that undergo ferroptosis in the heart.

Considering that many studies have successfully observed ferroptosis in animal models of multiple cardiomyopathy and that inhibition of ferroptosis by several methods can relieve cardiomyocyte injury, the major problem is how to immediately apply these findings to the diagnosis and treatment of cardiomyopathy in clinic. Combining existing information of antioxidant function with the growing mechanistic knowledge of ferroptosis to design novel approaches that may help in the identification and advancement of materials that can result in more specific methods to block iron-dependent ROS accumulation *in vivo*. While there are many obstacles to overcome, researching in the area has the possibility to illuminate new insights into cardiomyopathy and generate more efficient treatment modalities.

## Author Contributions

ZZ drafted the manuscript. ZZ, JL, PZ, FL, and ZX revised the manuscript. All authors have read and approved the final version of manuscript.

## Conflict of Interest

The authors declare that the research was conducted in the absence of any commercial or financial relationships that could be construed as a potential conflict of interest.
